# Optimization of treatment strategies for elderly patients with advanced non-small cell lung cancer

**DOI:** 10.3389/fonc.2024.1384906

**Published:** 2024-07-16

**Authors:** Qiang Chen, Shuo Ying, Jianwen Qin, Li Zhang

**Affiliations:** ^1^ Department of Respiratory and Critical Care Medicine, Tianjin Chest Hospital, Tianjin, China; ^2^ Department of Cardiology, Tianjin Chest Hospital, Tianjin, China

**Keywords:** advanced non-small cell lung cancer (NSCLC), elderly, lack of clinical evidence, assessment tools, optimized treatment

## Abstract

Lung cancer stands as a malignant neoplasm bearing the highest burden of morbidity and mortality within the elderly population on a global scale. Among the lung cancer subtypes, non-small cell lung cancer (NSCLC) prevails as the most prevalent. As age advances, elderly patients often present with an increased prevalence of comorbidities, diminished organ reserve function, and alterations in drug pharmacokinetics, including absorption, distribution, metabolism, and clearance. These factors collectively contribute to a reduction in their capacity to tolerate therapeutic interventions. Regrettably, there exists a paucity of research data and evidence regarding the management of elderly patients afflicted by advanced lung cancer. This article endeavors to compile and elucidate strategies for the enhancement of treatment approaches, with the aim of aiding clinical decision-making. Prior to the selection of clinical treatment modalities for elderly patients with advanced NSCLC, a comprehensive assessment should be conducted, taking into account various facets, including tumor characteristics, patient age, physiological status, and the presence of comorbidities. The treatment strategy should be implemented in a tiered fashion, thereby affording the opportunity for the tailoring of individualized therapeutic approaches for elderly patients afflicted by advanced NSCLC. The demographic of elderly patients confronting advanced NSCLC presents a complex landscape marked by intricate underlying conditions, necessitating the imperative optimization of treatment strategies.

## 1 Introduction

In 2020, lung cancer ranked as the second most frequently diagnosed malignancy and claimed the top spot as the leading cause of cancer-related mortality. It constituted roughly 11.4% of all newly diagnosed cancer cases and accounted for a staggering 18.0% of cancer-related deaths (1). In the year 2023, it is projected that approximately 350 individuals will succumb to lung cancer daily in the United States, firmly maintaining its status as the foremost cause of cancer fatality (2). This ailment predominantly affects the elderly population, with the median age at the time of diagnosis hovering around 70 years (3). Among the various forms of lung cancer, non-small cell lung cancer (NSCLC) prevails as the most prevalent, comprising approximately 85% of cases (4).

Elderly patients grappling with this disease often present with an array of underlying health conditions, utilize numerous concomitant medications, experience a decline in organ function, and undergo alterations in pharmacokinetics and pharmacodynamics. Paradoxically, this patient demographic is frequently underrepresented in clinical trials. Conventional lung cancer treatments may exacerbate the incidence of increasingly severe adverse events (AEs) in this context. The burgeoning field of geriatric oncology has witnessed significant advancements in recent years, advocating for a comprehensive evaluation of elderly individuals both before and during their cancer treatment, aiming to deliver more precise therapeutic interventions (5). The primary objective of this article is to consolidate and elucidate the concept of geriatric assessment and the optimization of treatment strategies for elderly patients with advanced NSCLC, with the aspiration of furnishing a valuable reference for clinical practice.

### 1.1 Definition of old age

The definition of ‘old age’ lacks a universally accepted standard due to its subjective nature, reliant upon social, economic, and health-related variables. In most industrialized societies, old age is conventionally defined at the age of 70, whereas in less affluent regions, age 65, 60, or even 55 might serve as the demarcation point (6). The National Comprehensive Cancer Network (NCCN) Geriatric Oncology Guidelines delineate the elderly as individuals aged 65 and above, further subdividing them into three categories: those aged 65 to 75 categorized as young elderly, those between 76 and 85 as elderly, and those over 85 as advanced aged (7).

## 2 Optimization strategy

### 2.1 Strategy 1: utilize appropriate tools for pre-treatment assessment

The elderly population exhibits a considerable degree of heterogeneity, with age alone unable to adequately capture the extent of aging. In the realm of geriatric oncology, treatment strategies for patients should pivot primarily on functional status rather than age, allowing for a balanced consideration of the benefits and risks associated with treatment. Therefore, a comprehensive assessment of the patient’s overall condition before initiating treatment is imperative to maximize organ function preservation during the therapeutic process (6).

Several assessment tools are currently employed to evaluate the health status of cancer patients, predict treatment efficacy, and assess tolerance. Karnofsky Performance Status (KPS) and Eastern Cooperative Oncology Group (ECOG) Performance Status (PS) scores are widely used to evaluate the functional status of cancer patients. However, these methods fall short in capturing the overall status of elderly cancer patients and accurately predicting adverse outcomes of chemotherapy, thereby having limitations in guiding treatment (8). Consequently, the International Society of Geriatric Oncology (SIOG) and the American Society of Clinical Oncology (ASCO) strongly advocate for the incorporation of comprehensive geriatric assessment (CGA) into the management plans for these patients. CGA encompasses multiple dimensions beyond conventional medical assessment, including functional status, fatigue, comorbidities, cognitive function, mental health, social support, nutrition, and geriatric syndromes (9, 10). A systematic review conducted by Hamaker et al. (11) revealed that 28% of patients modified their oncology treatment plans, with the majority receiving fewer intensive regimens, while a median of 72% of patients opted for non-oncological interventions. 75% of the studies in this review demonstrated that the geriatric assessment group exhibited higher treatment completion rates, with 55% of the studies indicating lower treatment-related toxicities or complications. Quite a few real-world studies use ECOG PS as an assessment tool, which limits the ability to generalize data and compare it with other case series from different institutions. Current studies suggest that age and PS scores do not fully reflect the physical condition of elderly patients, and that CGA should be conducted according to the guidelines to avoid overtreatment or undertreatment (12–16). It is inferred that geriatric assessment can enhance treatment tolerance and completion in elderly cancer patients.

#### 2.1.1 Chemotherapy risk assessment tools: cancer aging research group (CARG) and chemotherapy risk assessment scale for high-age patients (CRASH)

The primary tools recommended for assessing chemotherapy risk in elderly patients encompass the following: CARG chemotherapy risk assessment scale (17), CRASH (18), instrumental activities of daily living (IADL), activities of daily living (ADL), Charlson comorbidity index (CCI), cumulative illness rating scale-geriatric (CIRS-G), mini-mental state examination (MMSE), geriatric depression scale (GDS), geriatric screening tool-8 (G-8) and vulnerable elders survey-13 (VES-13), among others (8).

Of particular clinical significance, CARG and CRASH exhibit comprehensive coverage and robust clinical applicability. Moreover, they exhibit comparable predictive performance for chemotherapy resistance (19), positioning them as the most promising tools for optimizing chemotherapy regimens (6). Hurria et al. (17) initially introduced the CARG scale in a prospective cohort study involving 500 cancer patients aged 65 and older, with 29% diagnosed with lung cancer. The study found that patients classified as low risk, medium risk, or high risk based on the CARG scale had proportions of grade three to five chemotherapy-related AEs of 30%, 52%, and 83%, respectively (P < 0.001). Conversely, when risk grouping was based on KPS scores, no significant difference in the incidence of chemotherapy-related AEs was observed in each group (P = 0.19). Subsequent analysis involved calculating the area under the receiver operating characteristic (ROC) curve, revealing that the CARG outperformed KPS in predicting chemotherapy-related AEs (0.72 vs. 0.53). This has led to the speculation that the CARG scale possesses predictive capabilities regarding chemotherapy tolerance in elderly patients, a hypothesis substantiated by subsequent research (20). In 2012, Extermann et al. (18) proposed the CRASH scale for the first time. The scale was based on a introduced the CRASH scale, based on a prospective cohort study encompassing 562 cancer patients, including 518 evaluable cases, with an average age of 70 years or older (20% of whom were lung cancer patients). The study demonstrated that the CRASH scale could predict the incidence of hematological and non-hematological toxicity induced by chemotherapy drugs, suggesting its potential to forecast chemotherapy tolerance in elderly patients. CARG and CRASH are shown in **Tables 1**–**3**, respectively.

**Table 1:** CARG chemotherapy risk assessment scale.

Predictors		Points
Age (year)	65 to <72	0
	≥72	2
Cancer type	Other	0
	GI or GU	2
Chemotherapy dosing	Reduced	0
	Standard	2
No. of chemotherapy agents	Monochemotherapy	0
	Polychemotherapy	2
Hemoglobin (g/dL)	≥11 (male), ≥10 (female)	0
	<11 (male), <10 (female)	3
Creatinine clearance (mL/min)	≥34	0
	<34	3
Hearing	good	0
	fair or worse	2
No. of falls in last 6 months	None	0
	≥1	3
Medication intake	No assistance	0
	with some help/unable	1
Limited in walking 1 block	Not limited at all	0
	Somewhat limited/limited a lot	2
Decreased social activity because of health/emotional problems	A little, or none of the time	0
	Some, most, all of the time	1

CARG, Cancer and Aging Research Group; GI, gastrointestinal; GU, genitourinary; Low risk: 0-5 points, medium risk: 6-9 points, high risk: ≥10 points

**Table 2:** CRASH score.

Predictors	Points			Risks	
	0	1	2	Single	combined
Hematologic score^*^				Low: 0-1 points	Low: 0-3 points
Diastolic BP	≤72	>72		Med low: 2-3 points	Med low: 4-6 points
IADL	26-29	10-25		Med high: 4-5 points	Med high: 7-9 points
LDH (if ULN 618 U/L; otherwise, 0.74/L*ULN)	0-459		>459	High: 6-8 points	High: ≥10 points
Chemotox^&^	0-0.44	0.45- 0.57	>0.57		
Nonhematologic score^*^				Low: 0-2 points	
ECOG PS	0	1-2	3-4	Med low: 3-4 points	
MMS	30		<30	Med high: 5-6 points	
MNA	28-30		<28	High: 7-8 points	
Chemotox^&^	0-0.44	0.45-0.57	>0.57		

CRASH, the chemotherapy risk assessment scale for high-age patients; BP, blood pressure; Chemotox, toxicity of the chemotherapy regimen; ECOG PS, Eastern Cooperative Oncology

Group performance status; IALD, instrumental activities of daily living; LDH, lactate dehydrogenase; MMS, Mini Mental Health Status; MNA, Mini Nutritional Assessment; ULN, upper limit of normal.

^*^ For the combined risks, add the points from the hematologic and nonhematologic score, counting Chemotox only once.

^&^ For examples of Chemotox values for specific regimens, see **Table 3**.

**Table 3:** Example of chemotox values for various chemotherapy regimens.

Points^#^		
0	1	2
Capecitabine 2g	Capecitabine 2.5 g	5-FU/LV (Roswell-Park)
Cisplatin/pemetrexed	Carboplatin/gemcitabine AUC 4-6/1 g d1, d8	5-FU/LV (Mayo)
Dacarbazine	Carboplatin/pemetrexed	5-FU/LV and bevacizumab
Docetaxel weekly	Carboplatin/paclitaxel q3w	CAF
FOLFIRI	Cisplatin/gemcitabine d1, d8	Carboplatin/docetaxel q3w
Gemcitabine 1 g 3/4 wk	ECF	CHOP
Gemcitabine 1.25 g 3/4 wk	Fludarabine	Cisplatin/docetaxel 75/75
Paclitaxel weekly	FOLFOX 85 mg	Cisplatin/etoposide
Pemetrexed	Gemcitabine 7/8 wk then 3/4 wk	Cisplatin/gemcitabine d1, d8, d15
	Gemcitabine/irinotecan	Cisplatin/paclitaxel 135-24 h q3w
	PEG doxorubicin 50 mg q4w	CMF classic
	Topotecan weekly	Doxorubicin q3w
	XELOX	FOLFOX 100-130 mg
		Gemcitabine/pemetrexed d8
		Irinotecan q3w
		Paclitaxel q3w
		Docetaxel q3w
		Topotecan monthly

5-FU, 5-fluorouracil; AUC, area under the concentration-time curve; CAF, cyclophosphamide, doxorubicin,and 5-fluorouracil; CHOP, cyclophosphamide, doxorubicin, vincristine, and prednisone; CMF, cyclophosphamide, methotrexate, and 5-fluorouracil; ECF, epirubicin, cisplatin and 5-fluorouracil; FOLFIRI, irinotecan, leucovorin, and 5-fluorouracil; FOLFOX, folinic acid, 5-fluorouracil, and oxaliplatin; LV, leucovorin; PEG, pegylated; q3w, every 3 weeks; q4w, every 4 weeks; XELOX, capecitabine and oxaliplatin

^#^Unless specified otherwise, the doses are per meter squared. If no dose is specified, then this means that the various common doses used for this regimen all fall into the same category.

#### 2.1.2 Targeting and immunotherapy evaluation tools: G-8 and VES-13

The utility of CGA in guiding targeted and immunotherapy for elderly patients with advanced NSCLC remains an evolving field with no established assessment tool. A prospective observational cohort study by Gomes et al. (21) involved 140 elderly patients with cancer, of which 55% were diagnosed with NSCLC. The study categorized patients into elderly and young groups based on a 1:1 age ratio, with median ages of 75 and 62 years, respectively. The G-8 assessment was conducted before treatment in the elderly group, with a score of less than 15 indicating a positive result. Single-drug immune checkpoint inhibitors (ICIs) were administered as treatment. The study revealed that elderly patients with a positive G-8 assessment exhibited higher mortality and readmission rates, suggesting the G-8 score may play a role in predicting severe adverse events in frail elderly NSCLC patients. A recent review of screening assessment tools for elderly cancer patients (22) highlighted G-8 and VES-13 as the most commonly used assessment tools. G-8 demonstrated higher sensitivity, whereas VES-13 exhibited higher specificity, and both can be employed individually or in combination. However, it should be noted that these two assessment tools lack specificity for NSCLC, and there remains a dearth of high-quality research to validate their use.

However, CGA often requires multidisciplinary collaboration to accurately assess patients and is therefore very time-consuming, posing a significant barrier to its adoption in clinical practice (23). In the future, two approaches could be explored: First, the design of a more convenient evaluation tool, followed by large-scale prospective clinical trials to verify its effectiveness; second, the development of a calculator based on the current evaluation tool to facilitate the calculation of scores and assist in assessing pre-treatment risk.

### 2.2 Strategy 2: mitigate drug interactions

Elderly lung cancer patients often find themselves taking multiple medications to manage various comorbid conditions. Some studies (24, 25) have reported that the median number of concomitant medications for elderly cancer patients ranges from five to nine, with approximately 35% of patients experiencing significant drug interactions. A concise listing of common NSCLC treatment drugs and the potential effects of concurrent medications is provided for reference in **Table 4** (8).

**Table 4:** Common NSCLC treatment drugs have related effects with other drugs.

medicine	other drugs	result
Carboplatin, etoposide, gemcitabine, paclitaxel, and gefitinib	Warfarin	Increase the blood concentration of warfarin and the risk of bleeding
Cisplatin	Phenytoin	Reduce the blood concentration of phenytoin, which is not conducive to epilepsy control
First- and third-generation EGFR-TKIs	carbamazepine, phenytoin	Reduce the plasma concentration of first- and third-generation EGFR-TKIs and affect the efficacy
First-generation EGFR-TKIs	itraconazole	Increase the plasma concentration of first-generation EGFR-TKIs and increase adverse drug reactions
First-generation EGFR-TKIs	PPIs	Reduce the absorption of first-generation EGFR-TKIs and increase the risk of death
ICIs	PPIs	Affect the efficacy of ICIs and increase the risk of poor prognosis

NSCLC, non-small cell lung cancer; EGFR-TKIs, epidermal growth factor receptor-tyrosine kinase inhibitors; PPIs, proton pump inhibitors; ICIs, immune checkpoint inhibitors.

### 2.3 Strategy 3: tailor drug dosages based on liver and kidney function

Hepatic and renal insufficiency is prevalent among elderly lung cancer patients. Consequently, when administering anti-tumor drugs subject to hepatic and renal metabolism, it is imperative to make appropriate adjustments to the dosage to mitigate adverse effects. A succinct compendium of common NSCLC treatment drugs necessitating dosage adjustments is provided for reference in **Table 5** (8).

**Table 5:** Common therapeutic drugs for NSCLC requiring dose adjustment.

Reason for adjustment	Representative medicine
Dosage needs to be adjusted based on renal function	Cisplatin, carboplatin, pemetrexed, etoposide, and crizotinib
Mild to moderate hepatic insufficiency requires to adjust dose	Docetaxel, paclitaxel, nab-paclitaxel, gemcitabine, gefitinib, erlotinib, crizotinib, and brigatinib
Severe hepatic impairment requires dose adjustment	Alectinib, ceritinib, osimertinib, pemetrexed, etoposide, and vinorelbine

### 2.4 Strategy 4: selecting the optimal treatment option

Clinical trials provide a critical foundation for formulating guidelines and guiding treatment. However, current clinical trial results cannot be generalized to elderly patients with advanced NSCLC. Subgroup analyses of older patients were conducted retrospectively, and those who participated in clinical trials were generally healthier than those treated in routine practice, resulting in a lack of real-world evidence. Additionally, traditional cancer clinical trials are often time-consuming and expensive, and they frequently produce results with limited real-world applicability, posing challenges for patient participation.

Real-world data studies offer a promising solution to fill evidence gaps and provide essential information about the effects of cancer treatments in real-world settings. However, the quality of real-world data can affect the reliability of real-world evidence. Therefore, combining traditional clinical trials with real-world data studies can provide a stronger foundation for treatment decisions in elderly patients with advanced NSCLC (26).

#### 2.4.1 Preferred treatment for patients with positive driver mutations: targeted Therapy

The driver gene profiles of elderly patients exhibit certain characteristics, which, however, are not significantly different from those of younger patients. Targeted therapy offers distinct advantages, including minimal side effects, good tolerance, enhanced quality of life, and potential improvements in prognosis. Consequently, it is recommended that patients with non-squamous NSCLC and certain squamous cell carcinomas undergo routine screening for specific driver gene mutations, such as epidermal growth factor receptor (EGFR) mutations, anaplastic lymphoma kinase (ALK) fusion genes, ROS1 fusion genes, RET fusion genes, BRAF gene V600E mutation, MET gene exon 14 skipping mutation, and other pertinent driver genes. Targeted therapy is the primary treatment choice for elderly patients with advanced NSCLC who test positive for these driver mutations (8, 27).

##### 2.4.1.1 EGFR - tyrosine kinase inhibitors: third generation > second generation > first generation

In China, EGFR-TKIs approved for first-line treatment are categorized into three generations: the first generation includes gefitinib, erlotinib, and icotinib; the second generation comprises afatinib and dacomitinib, while the third generation features osimertinib and ametinib. A meta-analysis conducted by Greenhalgh et al. (28) revealed that when compared to chemotherapy, EGFR-TKIs demonstrate superior outcomes, including a better tumor response rate, extended progression-free survival (PFS), fewer AEs, and an enhanced health-related quality of life. However, it is noteworthy that limited research has indicated whether EGFR-TKIs contribute to longer overall survival (OS).

Meta-analyses have underscored the advantages of EGFR-TKIs in the treatment of elderly patients with advanced NSCLC. However, these studies have not delved into the therapeutic distinctions among various EGFR-TKIs. A retrospective observational cohort study comparing first- and second-generation EGFR-TKIs (29) among patients aged 60 years and older, it was found that the median OS was 19.1 months for gefitinib, 22.9 months for erlotinib, and an impressive 35.6 months for afatinib. The OS of the afatinib group not only exceeded that of the gefitinib group (P= 0.009) but also outperformed the gefitinib combined with erlotinib group (35.5 vs. 21.4 months, P=0.016). Remarkably, there was no statistically significant difference in PFS among these three groups. This suggests that the longer OS observed in the afatinib group might be attributed to different resistance mechanisms that manifest during treatment. Subgroup analysis from the successive ARCHER1050 studies (30, 31) demonstrated that dacomitinib can significantly prolong PFS compared with gefitinib in patients aged 65 years and older (Hazard Ratio (HR)= 0.69, 95% confidence interval (CI): 0.48-0.99), though there was no significant OS benefit (HR=0.987, 95% CI: 0.687-1.419).

It’s important to note that the selected population of these studies excluded individuals who had developed central nervous system (CNS) metastasis, a condition associated with shorter survival. Among NSCLC patients with EGFR mutations, roughly 25% present with CNS metastasis at the time of diagnosis, and approximately 50% develop CNS metastasis within three years of diagnosis (32).

Moreover, most NSCLC patients with EGFR mutations experience disease progression after nine to thirteen months, with over half attributed to the EGFR exon 20 T790M mutation (33). As a third-generation EGFR-TKI, osimertinib can selectively inhibit EGFR-TKI sensitizing mutations and T790M resistance mutations, while also exhibiting activity within the CNS. The FLAURA study (34, 35) confirmed that the use of osimertinib in patients aged 65 years and older could significantly extend PFS compared to first-generation EGFR-TKIs (HR=0.49, 95% CI: 0.35-0.67). However, the OS benefit was not statistically significant (HR=0.87, 95% CI: 0.63-1.22).

In the last five years, real-world studies have shown that although EGFR-TKIs are effective and safe for older adults, and their PFS in patients is generally consistent with the results of clinical trials, the improvement in OS is limited (3, 36, 37). One study found that older individuals treated with osimertinib had longer PFS than those treated with first-generation EGFR-TKIs. However, it cannot be ignored that osimertinib has a higher risk of pneumonia compared to first-generation EGFR-TKI therapy (38).

##### 2.4.1.2 ALK-TKIs: Alectinib as the preferred choice

ALK fusion gene positivity is a relatively rare occurrence in NSCLC, accounting for approximately 3 to 5% of cases. It is more prevalent among younger individuals, those with adenocarcinoma, and never-smokers. ALK-TKIs approved for use in China are categorized into two generations: the first generation, represented by crizotinib, and the second generation, which includes alectinib, ceritinib, and ensartinib. A subgroup analysis of the PROFILE 1014 study (39) revealed that elderly patients aged 65 years or older treated with crizotinib experienced longer PFS when compared to chemotherapy (HR=0.37, 95% CI: 0.17-0.77). However, the clinical application of crizotinib is limited due to the high incidence of secondary mutations in the ALK gene during its treatment. The ASCEND-4 study (40) demonstrated the potential of ceritinib to prolong median PFS in various subgroups, including elderly patients aged 65 years or older (HR=0.45, 95% CI: 0.24-0.86), when compared to chemotherapy. While second-generation ALK-TKIs have shown promising response rates and survival benefits (41), studies focused on elderly patients remain scarce, with most results arising from subgroup analyses. A multicenter, randomized, open-label phase III study (42) found that ensartinib significantly extended the median PFS compared to crizotinib, though no significant difference was observed in the PFS subgroup analysis of elderly patients aged 65 years or older. The ALEX study (43) demonstrated that the use of alectinib in elderly patients aged 65 years or older, when compared to crizotinib, significantly prolonged PFS (HR= 0.45, 95% CI: 0.24-0.87). A real-world retrospective study (44) encompassing 53 patients with ALK fusion gene-positive advanced NSCLC categorized into two age groups (<65 and ≥65 years) and treated with crizotinib, ceritinib, and alectinib respectively, found that age did not significantly impact PFS and OS in either group. Patients treated with alectinib exhibited the lowest incidence of AEs, with ceritinib showing the highest, and crizotinib falling in between. This suggests that in elderly advanced NSCLC patients with ALK fusion gene positivity, crizotinib, ceritinib, and alectinib offer similar efficacy but varying safety profiles. Alectinib stands out with a lower incidence of serious AEs and a reduced rate of treatment discontinuation, making it a promising first-line treatment option for elderly NSCLC patients with positive ALK fusion genes (8).

##### 2.4.1.3 Other genetic mutations

fFor other gene mutations with lower incidence rates, we will provide concise recommendations. Savolitinib is a suitable option for elderly patients who have progressed after platinum-based chemotherapy with MET exon 14 skipping mutation or those who cannot tolerate platinum-based chemotherapy (45). Crizotinib is an effective choice for elderly patients with a ROS1 fusion-positive gene (46). The combination of dabrafenib and trametinib is recommended for elderly patients with a BRAF V600E mutation (47). Platinib is a viable treatment for elderly patients with a positive RET fusion gene (48).

#### 2.4.2 ICIs: pembrolizumab single agent is preferred

ICIs have ushered in groundbreaking advancements in the treatment of advanced lung cancer, making them a focal point in the realm of lung cancer treatment. Subgroup analysis of KEYNOTE-024 study (49) revealed that among elderly patients with advanced NSCLC exhibiting high expression of programmed cell death ligand 1 (PD-L1) (TPS ≥50%) and lacking EGFR/ALK mutations, pembrolizumab was consistent with the overall population in extending OS and significantly outperformed chemotherapy (HR=0.64, 95% CI: 0.42-0.98). In a subgroup analysis of the EMPOWER Lung-01 study (50), elderly patients with advanced NSCLC and high PD-L1 expression experienced significant extensions in both OS and PFS when treated with cemiplimab compared to chemotherapy. A real-world study (51) involving 2049 patients who received ICIs demonstrated that elderly patients aged ≥75 years, after undergoing immune monotherapy, exhibited no significant difference in OS compared to patients aged 50-75 or <50 years. Both non-elderly and elderly patients benefited from PFS when platinum-based chemotherapy was combined with pembrolizumab in the Keynote-189 (52) and Keynote-407 (53), though the benefit was somewhat lower in elderly patients. In the IMpower 150 study (54), elderly patients aged ≥75 years did not experience a significant PFS benefit with atezolizumab plus bevacizumab plus carboplatin plus paclitaxel (ABCP) compared to the bevacizumab plus carboplatin plus paclitaxel (BCP) group, while non-elderly patients showed significant benefits in a subgroup (65-75 years old: 9.7 vs 6.9 months, P<0.05; <65 years old: 8.0 vs 6.8 months, P<0.05). In the phase III randomized CheckMate-227 trial (55), nivolumab combined with ipilimumab offered a modest OS benefit to patients aged ≥75 and 65-74 years old compared to chemotherapy, but this benefit was less pronounced than in patients under 65. In the Check-Mate 9LA study (56), patients aged ≥75 years did not derive an OS benefit, while those under 75 experienced significant OS benefits. These results suggest that the diminished OS benefit in elderly patients under intensive combination therapy may be associated with lower tolerability. According to the FDA’s retrospective summary analysis (57), when PD-L1 expression is ≥50%, there is no difference in survival between chemotherapy combined with ICIs and ICIs alone in patients aged 65-74 years. Patients aged ≥75 years exhibited better survival outcomes with ICIs than with chemotherapy combined with ICIs. For patients with PD-L1 expression of 1-49%, chemotherapy combined with ICIs was superior to ICIs alone in patients under 75 years old, but there was no difference in survival between these two treatment strategies in patients aged ≥75 years.

A meta-analysis (58) of patients receiving nivolumab for advanced renal cell carcinoma, melanoma, and NSCLC demonstrated that the incidence of all-grade AEs was similar in elderly and non-elderly patients, but elderly patients had a higher incidence of ≥grade three AEs (71.7% vs. 58.4%). Conversely, in a pooled analysis (59) encompassing the CheckMate-057, KEYNOTE-010, OAK, and POPLAR studies, the incidence of grade three to four immune-related AEs in individuals aged ≥75 years was lower than in each age group under 75 years (23% vs. 47%, 49%), and the incidence of AEs leading to treatment discontinuation was similar (5% vs. 7%, 7%). These findings suggest that older age does not increase the number of immune-related AEs leading to treatment termination and may even reduce it.

Although the real-world study included a heterogeneous population of patients treated with different types of PD-(L)1 inhibitors, these patients received different treatment regimens (60), and direct comparisons between the study results and clinical trials are not reasonable (61). However, real-world studies have reached conclusions similar to clinical studies, namely that old age is not a substitute for clinical frailty, nor is age a limiting condition for immunotherapy (12, 13, 23, 60, 62–75). Many studies have shown that older patients exhibit similar efficacy and safety in immunotherapy as the general population. A real-world study comparing the effectiveness of pembrolizumab, nivolumab, and atezolizumab found objective response rates (ORR) and disease control rates (DCR) of 22.4%, 8.2%, and 4.3% (p = 0.004) and 59.2%, 55.7%, and 30.0% (p = 0.001), respectively. Although there was no difference in OS between the three groups (12.6 months vs. 8.4 months vs. 7.7 months, p = 0.334), pembrolizumab had the longest OS. In the PD-L1 ≥ 50% subgroup, pembrolizumab showed a statistically significant OS advantage compared to atezolizumab (pembrolizumab vs. atezolizumab, p = 0.023; nivolumab vs. atezolizumab, p = 0.153; pembrolizumab vs. nivolumab, p = 0.406) (61).

In conclusion, it is recommended that elderly patients with advanced NSCLC who exhibit high PD-L1 expression should be treated with ICIs monotherapy as the first-line approach. While ICIs combination therapy demonstrates a beneficial trend in patients under 75 years old, there is insufficient evidence to support its use in patients aged ≥75 years.

#### 2.4.3 Chemotherapy: preferential use of single-agent regimen with third-generation non-platinum chemotherapy drugs for patients lacking driver genes or exhibiting low PD-L1 expression in NSCLC

The third generation of non-platinum chemotherapy drugs comprises agents such as vinorelbine, gemcitabine, paclitaxel, docetaxel, and pemetrexed. Previous studies have extensively examined the survival outcomes and safety profile of chemotherapy in elderly lung cancer patients. For elderly patients with advanced NSCLC who lack targeted driver gene mutations and exhibit low PD-L1 expression, platinum-containing doublet combination therapy is the recommended first-line treatment option for those who are suitable (76). However, this approach can be associated with greater AEs, making it unsuitable for elderly patients or individuals in poor health. The ELVIS study (77) investigated 191 elderly patients aged 70 years and above with advanced NSCLC. Results revealed that, when compared to the best supportive care (BSC) group alone, the vinorelbine combined with BSC group significantly prolonged the median survival time (MST) (28 weeks vs. 21 weeks), improved the 1-year survival rate (32% vs. 14%), and enhanced the quality of life (QOL). A meta-analysis (78) that included data from 10 studies involving a total of 2,510 elderly patients with advanced NSCLC demonstrated that the response and survival rates were superior in the platinum-containing doublet chemotherapy group compared to single-agent therapy. However, it’s worth noting that the incidence of grade 3/4 adverse events such as anemia, thrombocytopenia, and neurological toxicity was higher in the doublet chemotherapy group.

A real-world study involving 474 consecutive elderly patients (≥70 years of age) diagnosed with stage IIIB-IV NSCLC found that a platinum-based dual-drug regimen (OR 2.23, 95% CI 1.02-4.87, p<0.04) was an independent risk factor for hospitalization. The use of a platinum-based dual-drug regimen was associated with a higher risk of hospitalization and conferred no survival benefit compared to a third-generation single-drug chemotherapeutic regimen (79).

In summary, when considering treatment options for elderly patients, it is crucial to conduct a comprehensive assessment of their overall health and ability to tolerate double-drug chemotherapy. This approach is recommended as the first-line treatment for elderly patients without driver gene mutations and with low PD-L1 expression.

#### 2.4.4 Anti-angiogenic drugs: consistency in therapeutic dosage and safety across the patient population

Anti-angiogenic therapeutic drugs, whether administered alone or in combination with chemotherapy, EGFR-TKIs, or immune checkpoint inhibitors, have demonstrated significant efficacy (8). The ALTER0303 study (80) revealed that anlotinib exhibited notable benefits for elderly patients, exhibiting superior PFS (HR=0.22, 95% CI: 0.07-0.64) and OS (HR=0.34, 95% CI: 0.12-0.94), particularly among those aged ≥70 years. Conversely, the POINTBREAK study (81) showed that while the combination of chemotherapy with anti-angiogenic drugs extended PFS compared to chemotherapy alone (6.0 months vs. 5.6 months), there was no significant difference in OS. The ARIES study (82) reported that combining bevacizumab with chemotherapy in elderly patients did not result in different PFS and adverse event profiles when compared to their non-elderly counterparts, although OS was slightly shorter. In the NEJ026 study (83), elderly patients with EGFR fusion gene-positive NSCLC, both those < 75 and ≥75 years old, experienced PFS benefits from erlotinib combined with bevacizumab. Similarly, the ACTIVE study (84) demonstrated improved PFS in the elderly subgroup when apatinib was combined with gefitinib (HR=0.9 vs. 0.67). Studies like those referenced (82, 85, 86) indicate that the adverse event grading for bevacizumab combined with chemotherapy mostly remained below grade two, with no statistical difference in the incidence of grade three and higher adverse events between elderly and non-elderly patients. This suggests that the safety profile of anti-angiogenic treatment is comparable for both elderly and non-elderly lung cancer patients.

In a real-world study that retrospectively collected electronic medical records of NSCLC patients receiving Endostar combined with chemotherapy, 554 and 571 patients were assigned to ≤60 years of non-elderly patients and >60 years of elderly patients, respectively, and performed propensity score matching. Results showed no significant difference in efficacy between the two groups, and the adverse reactions were tolerable (87). Another study retrospectively enrolled 83 elderly patients (>65 years of age) with NSCLC who had previously received at least two lines of systemic therapy and whose disease had progressed. The ORR was 7.2% (95% CI = 2.7-15.1%) and the DCR was 78.3% (95% CI = 67.9-86.6%), consistent with the ALTER0303 clinical trial. This study found that the third-line efficacy of anlotinib monotherapy in the treatment of elderly patients with advanced NSCLC was satisfactory, and the safety was tolerable (88).

It is important to note that elderly patients often present with underlying cardiovascular and cerebrovascular conditions, and the risk of these conditions may increase with the use of anti-angiogenic drugs. Therefore, treatment decisions should not be based solely on age and should be approached with caution and vigilant monitoring.

#### 2.4.5 Radiotherapy - dearth of robust evidence presently

For patients with unresectable stage III NSCLC, the guidelines recommend concurrent chemoradiotherapy (cCRT) with subsequent durvalumab treatment for one year (76). Subgroup analysis of the PACIFIC study (89, 90) compared patients who received cCRT followed by durvalumab with those who received cCRT followed by a placebo. In the elderly subgroup aged ≥65 years, there was a prolonged PFS (HR=0.74, 95% CI: 0.54-1.01) and a 5-year OS (HR=0.79, 95% CI: 0.60-1.05), although the differences were not statistically significant. A retrospective study conducted using real-world data from the Netherlands (91) involved 2,942 patients with stage III NSCLC who underwent radical chemoradiotherapy (CRT). The study categorized patients into two groups: cCRT and sequential chemoradiotherapy (seqCRT). The median ages for these groups were 66 and 69 years, respectively. The study found that age itself was not a risk factor for acute toxicity or 3-month mortality after a three-month follow-up. However, it was noted that patients treated with cCRT, those with a higher TNM stage (IIIC) and poorer baseline health status had significantly higher three-month toxicity.

A retrospective analysis was conducted in patients with unresectable lung cancer who received treatment. Although older patients who received synchronous CRT had better OS (median OS: 40.9 months vs. 24.4 months), this difference was not statistically significant in the multivariate analysis (P = 0.09), suggesting that the treatment outcome in the elderly remained unsatisfactory and that the effect of multimodal therapy on elderly patients was limited (92). Two other studies found no association between age 70 and factors such as grade 3-4 CRT or Durvalumab toxicity, reduced chemotherapy dose, delay or cessation of treatment, progression, or death. These findings reinforce the current guideline recommendation that cCRT is associated with optimal outcomes in unresectable locally advanced NSCLC, even in older patients (93, 94).

In summary, there is currently insufficient evidence to make strong recommendations regarding the use of radiotherapy and chemotherapy in elderly patients with stage III NSCLC.

#### 2.4.6 Surgical interventions: current lack of sufficient evidence

The current guidelines (76) do not provide a surgical strategy for elderly patients with advanced NSCLC, and the suitability of surgical interventions for such patients remains undetermined. Kirk et al. (95) conducted a retrospective study to investigate the safety of lobectomy in NSCLC patients aged 80 years or older. They found that surgical morbidity and mortality were not increased in this age group; however, it’s important to note that the proportion of patients in this age category was low (4.9%). Additionally, these patients underwent rigorous screening and had low rates of smoking and pre-existing respiratory, cardiovascular, and neurological diseases. These factors could potentially introduce biases into the conclusions. As a result, more prospective research evidence is necessary to establish whether elderly patients with advanced NSCLC can benefit from surgical interventions.

## 3 Conclusions

the incidence of lung cancer in the elderly is on the rise, and these patients often present complex underlying health conditions. The available clinical evidence for guiding treatment decisions is notably limited, making the precise treatment of elderly patients a significant challenge. While some assessment tools for elderly patients are currently used in clinical practice, their results and simplicity are not ideal. These tools are primarily geared towards making chemotherapy decisions, and there remains a notable absence of tools designed for targeted therapies and immunotherapies.

For elderly patients with advanced NSCLC who possess driver genes, targeted therapy is the preferred treatment, though its efficacy might be reduced in patients with an ECOG PS score of two or higher. The G-8 and VES-13 scales are useful for pre-treatment evaluation. When chemotherapy is the chosen treatment for elderly patients with advanced NSCLC, the CARG or CRASH scale can be employed to assess their chemotherapy tolerance before initiating treatment. Elderly patients with advanced NSCLC and high PD-L1 expression can receive immune monotherapy, but combination therapy is not recommended for those aged 75 and older. Anti-angiogenic drugs can be used either alone or in combination and have demonstrated effectiveness in elderly patients with advanced NSCLC, but a thorough assessment of the risks related to blood and cerebrovascular diseases is essential.

Furthermore, elderly patients face numerous unfavorable factors when it comes to treatment, and distinguishing whether their death is due to cancer or other causes can be challenging. Therefore, the primary focus should be on preserving or enhancing their quality of life and functional status, with extending overall survival being a secondary objective.

**Figure 1:**
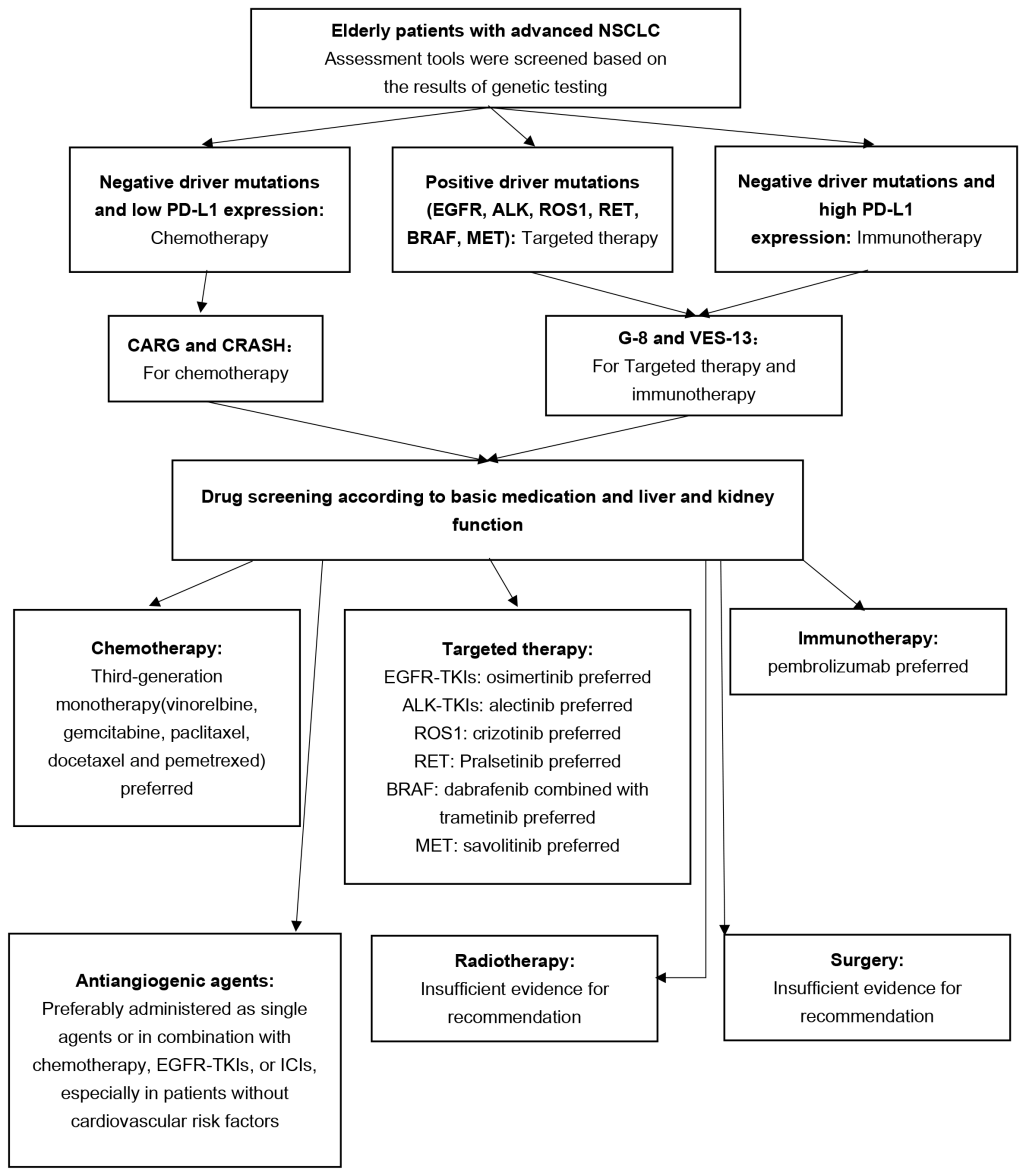
Flowchart for treating advanced non-small cell lung cancer in the elderly.

In the future, it is imperative to develop more straightforward and accurate assessment tools and include a greater number of elderly patients in prospective clinical studies. This will provide stronger evidence support for future treatment options and help address the unique challenges associated with treating elderly patients with lung cancer (**Figure 1**).

## Author contributions

QC: Formal analysis, Methodology, Visualization, Writing – original draft. SY: Data curation, Investigation, Writing – original draft. JQ: Validation, Visualization, Writing – original draft. LZ: Conceptualization, Supervision, Writing – review & editing.
